# Longitudinal Relationship between Personal CO and Personal PM_2.5_ among Women Cooking with Woodfired Cookstoves in Guatemala

**DOI:** 10.1371/journal.pone.0055670

**Published:** 2013-02-26

**Authors:** John P. McCracken, Joel Schwartz, Anaite Diaz, Nigel Bruce, Kirk R. Smith

**Affiliations:** 1 Department of Environmental Health, Harvard School of Public Health, Boston, Massachusetts, United States of America; 2 Center for Health Studies, Universidad del Valle de Guatemala, Guatemala City, Guatemala; 3 Department of Public Health and Policy, University of Liverpool, Liverpool, United Kingdom; 4 School of Public Health, University of California, Berkeley, California, United States of America; University of California San Francisco, United States of America

## Abstract

Household air pollution (HAP) due to solid fuel use is a major public health threat in low-income countries. Most health effects are thought to be related to exposure to the fine particulate matter (PM) component of HAP, but it is currently impractical to measure personal exposure to PM in large studies. Carbon monoxide (CO) has been shown in cross-sectional analyses to be a reliable surrogate for particles<2.5 µm in diameter (PM_2.5_) in kitchens where wood-burning cookfires are a dominant source, but it is unknown whether a similar PM_2.5_-CO relationship exists for personal exposures longitudinally. We repeatedly measured (216 measures, 116 women) 24-hour personal PM_2.5_ (median [IQR] = 0.11 [0.05, 0.21] mg/m^3^) and CO (median [IQR] = 1.18 [0.50, 2.37] mg/m^3^) among women cooking over open woodfires or chimney woodstoves in Guatemala. Pollution measures were natural-log transformed for analyses. In linear mixed effects models with random subject intercepts, we found that personal CO explained 78% of between-subject variance in personal PM_2.5_. We did not see a difference in slope by stove type. This work provides evidence that in settings where there is a dominant source of biomass combustion, repeated measures of personal CO can be used as a reliable surrogate for an individual's PM_2.5_ exposure. This finding has important implications for the feasibility of reliably estimating long-term (months to years) PM_2.5_ exposure in large-scale epidemiological and intervention studies of HAP.

## Introduction

Household air pollution (HAP) from use of solid fuels is estimated to be a major risk factor for diseases, including acute respiratory, chronic respiratory, cancer, and cardiovascular outcomes [1], [2]. Most of the epidemiological evidence for these relationships comes from studies using categorical exposure assignments based on stove and fuel types, which does not allow exposure-response analyses and limits comparability between studies in different settings. An ideal study design would include personal measures of exposure to the component of HAP that is causally related to the health effects being investigated. Fine particulate matter (PM_2.5_) is often considered the best pollutant to measure for studies of health effects from combustion-generated pollutant mixtures, including HAP, secondhand tobacco smoke, and ambient air pollution [3], [4]. Because of the size and weight of the monitoring equipment that has been available, personal PM measurements are generally burdensome and for infants infeasible, a particularly important limitation given the importance of quantifying the exposure-response relationship between HAP and pneumonia during infancy [5].

To overcome this problem, some HAP epidemiological studies have used area measurements of pollutant concentrations as surrogates for personal exposures. Kitchen area measures have been found to be poor surrogates of personal exposures to HAP [6]–[8], which may be largely attributable to differences in people's time-location patterns and the wide variability across small distances within the household and over short time periods [9]. Indirect exposure assessment, using time-activity patterns combined with area measurements [9], [10], may improve exposure assessment, but one study with simultaneous personal exposure measures indicated that this method has low validity [8].

An alternative approach to HAP exposure assessment is personal measurement of a surrogate pollutant for PM, such as carbon monoxide (CO), which is relatively easy and inexpensive to measure, for example with very small passive dosimeter tubes that can be attached to an infant's clothing. Both pollutants are products of incomplete combustion and are major components of biomass smoke [11]. Strong correlation has been found between CO and fine PM levels in kitchens where biomass fuels are used for cooking [12]–[14]. It has been unknown, however, whether the relationship between these pollutants in a fixed location can be extrapolated to personal exposures. Additionally, the aim of most HAP epidemiological studies is to investigate effects of long-term (several months to years) exposures, whereas the relationships between CO and PM have previously been evaluated only in cross-sectional designs [12], [14] or analyses [13].

The RESPIRE (Randomized Exposure Study of Pollution Indoors and Respiratory Effects) trial in Guatemala, the first randomized trial of an HAP exposure-reduction intervention, a chimney woodstove [5], for the prevention of pneumonia, included personal exposure measurements among a subset of women living in the study households. This short note presents a longitudinal analysis of the relationship between personal CO and PM among these women.

## Methods

The study population and exposure assessment methodology have been described previously [15], [16]. Briefly, women ≥38 years of age living in households participating in RESPIRE were recruited for a cardiovascular study. The study villages are located in the San Marcos department at approximately 2600 meters elevation above sea level. Smoking is uncommon, automobile traffic is low, and study households used only biomass fuels for cooking. The exposure assessment included a gravimetric (pump flow rate at 1.5 liters/minute, BGI Inc. sharp-cut cyclone inlet, 37 mm Teflon filter weighed before and after) measure of 24-hour personal exposure to particles with median aerodynamic diameter<2.5 µm (PM_2.5_). Simultaneously, continuous measurement of personal CO was performed with the span-gas calibrated Hobo (Onset Inc.) passive electrochemical datalogger, with conversion of CO ppm values to mass concentration for comparison with the PM mass concentrations [17]:
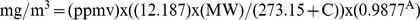
(1)where the molecular weight (MW) of CO is 28.01,

C, the mean temperature at the site, is 12 deg celsius

A is the elevation of each house in 100 meters (range 2250–2960 m)

We analyze measures (up to three per subject) taken during the trial period, when the intervention group used the chimney stove and the control group used the open fire for cooking.

Pollution measures were right-skewed, so we applied a natural log transformation to the data before assessing the relationship between personal CO and personal PM_2.5_ by scatterplot, correlation coefficients, and regression models. We used linear mixed effects models with personal PM_2.5_ as the dependent variable and random subject intercepts to account for correlation among repeated measures within subjects and to estimate the within- and between-subjects variance components. The model residuals were consistent with being derived from a normal distribution. We compared the variance of the random subject intercept between models to measure the extent to which between-subjects differences in typical personal PM_2.5_ are explained by covariates (R^2^_between_). For example, we estimated the R^2^_between_ for a model with CO as the independent variable by calculating the proportional reduction in the variance of the random subject intercept compared to the null model (no independent variable). The fixed effects in these models can be used to estimate personal PM_2.5_ based on covariates (stove, personal CO). To test for differences in the slope of PM_2.5_ on CO by stove type, we added a stove-by-CO interaction term. We tested for nonlinearity using a penalized spline for CO in a generalized additive mixed model (R software, GAMM function).

Protocols were approved by the Comité de Ética de la Universidad del Valle de Guatemala and the Harvard School of Public Health, Office of Human Research Administration. Written consents were obtained from all participants.

## Results

**Figure 1:**
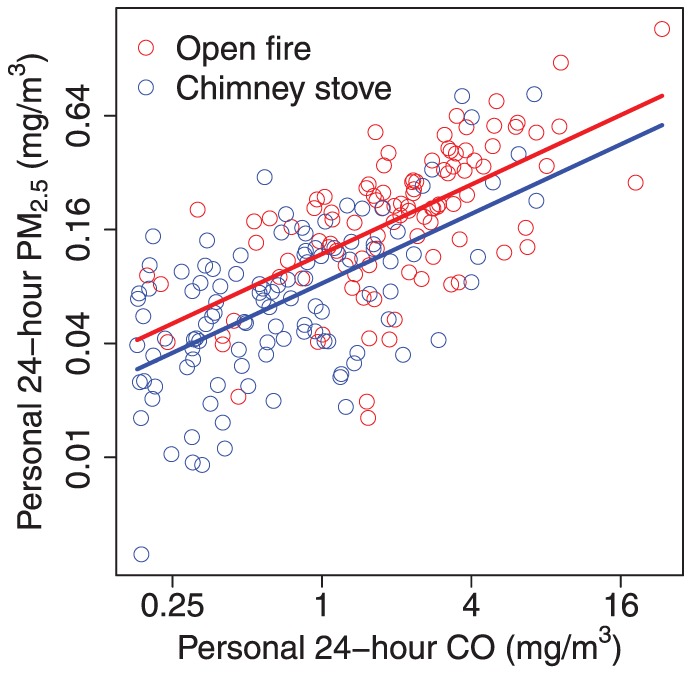
Scatter plot of simultaneous 24-hour personal fine particles (PM_2.5_) and personal carbon monoxide (CO). Lines for each stove type (red for open fire, blue for chimney stove) using equation PM_2.5_ = e^(−2.13+0.61*ln(CO) − 0.36*chimney)^ estimated from linear mixed effects regression model with natural log-transformed exposures (216 measurements among 116 women).

We obtained 216 simultaneous 24-hour measures of CO and PM_2.5_ among 116 women, 40 on one occasion, 52 on two occasions, and 24 on three occasions. The median (interquartile range) personal PM_2.5_ was 0.20 mg/m^3^ (0.11, 0.32) in the open fire group (67 women, n = 104) and 0.07 mg/m^3^ (0.04, 0.12) in the chimney stove group (49 women, n = 112), and personal CO was 2.02 mg/m^3^ (1.20, 3.35) in the open fire group and 0.63 mg/m^3^ (0.33, 1.22) in the chimney stove group. Figure 1 shows a direct relationship between the natural log-transformed values of personal CO and PM_2.5_ exposures. The Spearman rank correlation coefficient was 0.70 (p-value<0.001) between these two pollutant exposures (see Table 1).

**Table 1:** Effect estimates (95% confidence intervals) and variance components from linear mixed effects models to predict natural log personal PM_2.5_ (216 24-hour exposure measures among 116 subjects).

Independent variables	Chimney stove Effect	CO slope (per log-unit)	Between-subject variance	Within-subject variance	R^2^_between_
Null			0.31	0.76	
CO		0.69	0.07	0.48	0.78
		(0.59, 0.79)			
Stove type	−1.00		0.07	0.75	0.77
	(−1.25, −0.74)				
Stove type and CO	−0.36	0.61	0.05	0.48	0.85
	(−0.59, −0.12)	(0.50, 0.72)			
Plus stove by CO	−0.36	0.61	0.05	0.48	0.85
interaction*	(−0.60, −0.12)	(0.45, 0.77)			

* Stove by CO interaction effect = −0.00 (−0.22, 0.22).

In linear mixed effects models, the variance of the random intercept decreased from 0.31 to 0.07 when CO was added as an independent variable, equivalent to an R^2^_between_ = 0.78. A further reduction in random between-subject variability to 0.04 was achieved when stove type (chimney stove versus open fire) was added to the model (R^2^_between_ = 0.85).

The estimated population-mean personal PM_2.5_ based on personal CO alone can be calculated with the following equation:
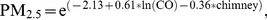
(2)where chimney = 1 for the chimney stove and chimney = 0 for open fire.

We did not find evidence of a difference in the slope by stove type (interaction p-value = 0.986), and we also did not find evidence of nonlinearity in these log-transformed data using generalized cross validation, which chose a spline with one degree of freedom (Figure 1).

## Discussion

Absent or minimal assessment of exposure to combustion-generated PM has been a major weakness of most epidemiological studies of HAP in developing countries, particularly those with the additional challenges presented by assessing these exposures among infants. Previous studies have shown that CO is strongly correlated with PM_2.5_ in kitchens where there is a single major source of smoke, but it was unclear whether this relationship could be extrapolated to personal exposures. We performed a longitudinal analysis of personal exposures among women from households in the RESPIRE trial in Guatemala, and found a moderately, strong correlation between personal CO and personal PM_2.5_. Repeated personal CO levels explain 78% of the between-subject variability in personal PM_2.5_. The estimated slope for the relationship between log-transformed measures is a 61% increase in personal PM_2.5_ per 100% increase in personal CO.

Our results contrast with those from a study conducted among children <5 years of age in the Gambia by Dionisio et al [18], who did not find evidence of correlation between personal CO and personal PM_2.5_ (r = −0.04), but there are a number of potential explanations for the weak correlations. In that population, there were a mixture of wood and charcoal stoves in use, which have substantially different ratios of CO to PM_2.5_ in their emissions [19], which would reduce the local correlation between personal PM_2.5_ and personal CO [9]. Similarly, the peri-urban children in the Gambian study may have been exposed to high levels of traffic emissions, with an even greater difference in CO:PM_2.5_ in its emissions [20]. Moreover, the durations of personal PM_2.5_ (48 hours) and personal CO (72 hours) measures differed in The Gambian study, which is expected to lower the correlation because of day-to-day variability in exposure levels. Finally, it is possible that instrument measurement error may have led to underestimation of the true correlation. Duplicate measures of each pollutant in a subset of participants should be collected in future studies to account for measurement error. Alternatively, longitudinal analyses can be used to separate within-subject variation (both true changes over time and measurement error) from between-subjects variation, as herein presented.

The form of the relationship between personal CO and personal PM_2.5_ in our models suggests a smaller increment of PM_2.5_ per unit of CO at higher exposure levels. This contrasts with the constant slope on the linear scale reported previously for kitchen concentrations in Guatemala [12]–[14], and there are several reasons why personal measurements may exhibit this relationship. Since combustion-generated PM_2.5_ is an irritant and the PM_2.5_-CO emissions ratio varies throughout the solid fuel burn cycle [11], it is possible that avoidance of the discomfort of PM and associated irritating compounds in the smoke by the householders may decrease the PM_2.5_-CO slope at higher exposure levels. In addition, particles tend to adhere to surfaces over time whereas CO does not. If people tend to be in the kitchen more after emissions have been exposed to surfaces around the household, this would also decrease the PM_2.5_-CO slope. Finally, the PM_2.5_-CO relationship may be different in microenvironments where people spend time other than the kitchen and the relative contribution of each microenvironment may vary by total exposure level.

## Conclusions

Our findings demonstrate that personal CO, which is relatively inexpensive and easy to measure can be a reliable surrogate for personal PM_2.5_ in some settings. We emphasize that the association was observed among women living in Guatemalan villages with a single dominant source of combustion, but may be modified by time-activity patterns associated with demographic characteristics, and is unlikely to be generalizable to settings with mixtures of pollution source types.
